# On-chip electrocatalytic microcells for high-throughput experimentation of electrocatalysts

**DOI:** 10.1016/j.isci.2026.116471

**Published:** 2026-06-30

**Authors:** Zerui Liu, Junxin Tao, Guojun Ou, Rui Cao, Chengyu Liu, Lin Jiang

**Affiliations:** 1 School of Microelectronics, Shanghai University, Shanghai 201800, China; 2 School of Chemistry and Chemical Engineering, Shaanxi Normal University, Xi’an 710119, China

**Keywords:** catalysis, electrochemistry, electrochemical materials science, materials science

## Abstract

On-chip electrocatalytic microcells (OCEMs) integrate working electrodes, reaction windows, and signal readout, offering structurally controllable and electronically addressable platforms with potential for high-throughput electrocatalysis. OCEMs enable precise control of catalyst features, including active sites, phase states, and defects, while tracking structural evolution and reaction intermediates in situ/operando. Coupling local current, electric fields, temperature, and strain modulation, OCEMs actively regulate catalytic behavior beyond conventional electrochemical measurements. When combined with automated high-throughput testing and machine learning, they generate interpretable and quantitative datasets, establishing clear structure-performance relationships. Together, these advances point toward an emerging OCEM-based high-throughput experimentation paradigm for data-driven, mechanism-informed catalyst design.

## Graphical abstract

**Figure:**
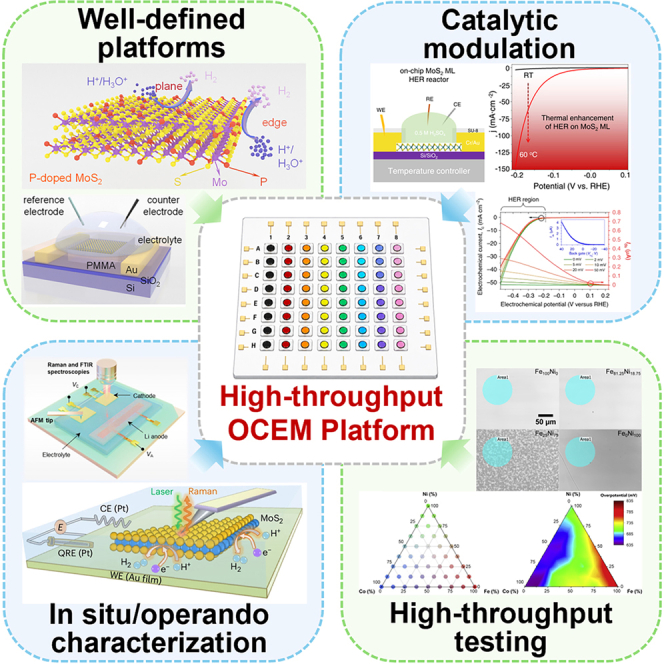

## Introduction

As a fundamental technology for sustainable energy conversion, electrocatalysis is undergoing a paradigm shift driven by the global push toward carbon neutrality. To address the intrinsic kinetic limitations of electrocatalytic reactions, the field has increasingly focused on the rational design of catalysts, in which structural morphology and surface reactivity are synergistically engineered to optimize catalytic performance.^1^^,^^2^^,^^3^^,^^4^^,^^5^^,^^6^^,^^7^^,^^8^^,^^9^ However, conventional catalysts often possess complex and heterogeneous structures, with non-uniform morphology, particle size, elemental distribution, and surface environment. This structural complexity severely complicates the establishment of unambiguous structure-performance relationships.^1^^,^^10^^,^^11^ The traditional catalyst development follows a linear, trial-and-error cycle encompassing material selection, synthesis, characterization, performance testing, and iterative optimization. Once target performance remains unmet, the entire process restarts from the beginning, leading to a time-consuming and inefficient research loop.^12^^,^^13^^,^^14^ These limitations impede the rational design of advanced electrocatalysts, underscoring the demand for accelerated discovery strategies, such as high-throughput screening.^15^^,^^16^^,^^17^^,^^18^

To date, automated testing platforms, combinatorial materials libraries, and array-based measurement techniques have allowed researchers to rapidly explore vast compositional and structural spaces with reduced manual effort and improved experimental throughput.^19^^,^^20^ More importantly, high-throughput strategies enable systematic statistical analysis and machine-learning (ML)-assisted predictive modeling.^21^^,^^22^ As a result, electrocatalyst discovery is gradually transitioning from heuristic trial-and-error toward integrated workflows that combine rapid experimentation, quantitative data acquisition, and model-guided rational design.^19^^,^^23^ However, for high-throughput experimentation (HTE) to evolve beyond rapid screening and enable mechanism-driven catalyst discovery, it must rely not only on measurement speed but also on high-quality, information-rich, and consistent datasets that support the extraction of fundamental physical and chemical principles.^10^^,^^24^ Beyond throughput, the underlying experimental platform must also provide precise structural control, array compatibility, and scalable fabrication.^25^^,^^26^

Over the past decades, on-chip electrocatalytic microcells (OCEMs) have emerged as a versatile experimental platform for HTE research.^25^^,^^27^ OCEMs are microdevices that integrate at least one working electrode and a spatially confined reaction window on a solid support. By integrating catalysts, electrodes, reaction windows, and signal readout within a micro/nanoscale device architecture, OCEMs provide well-defined, spatially confined, and electronically addressable model systems for HTE.^28^^,^^29^ Their open device configuration also enables seamless coupling with a suite of in situ and operando characterization techniques, including electrical transport measurements, Raman spectroscopy, infrared spectroscopy, mass spectrometry, and scanning probe-based methods.^24^^,^^30^ This compatibility allows real-time correlation of local structure dynamics, transient reaction intermediates, and catalytic performance.^10^^,^^30^ Accordingly, OCEMs are uniquely suited to identify catalytically active sites and track dynamic structural evolution under in situ/operando conditions. Meanwhile, they enable systematic investigations of structure-performance relationships while satisfying the standardization, array compatibility, and scalability required for practical HTE.^10^^,^^26^

Although OCEM research and high-throughput electrocatalysis share common goals, systematic integration of these two fields remains limited. Existing OCEM reviews primarily focus on device design and single-device studies, whereas HTE reviews emphasize combinatorial screening and automation. This review bridges this gap by positioning OCEMs as integrated platforms that enable structurally well-defined catalyst design, mechanistic understanding, and HTE. These platforms generate descriptor-rich and interpretable datasets beyond conventional approaches. We first outline how OCEMs serve as well-defined model systems for probing structure-performance relationships. We then discuss their unique ability to regulate charge transport, local reaction microenvironments, and catalytic behavior through microcell design. Next, we highlight the integration of OCEMs with in situ/operando characterization for mechanistic studies of dynamic structural evolution and active-site catalytic behavior. Finally, we emphasize the promising potential of OCEMs as scalable, array-compatible platforms for HTE and data-driven electrocatalyst discovery. This review seeks to clarify the value of OCEMs as a high-throughput experimental strategy and a reliable tool for mechanistic interpretation and rational catalyst design. It should be noted that most OCEM studies to date remain at the single-device proof-of-concept level, and their integration into truly high-throughput workflows is still in its infancy. Nevertheless, their inherent architectural advantages position them as promising candidates for potential HTE platforms, provided that critical challenges in fabrication consistency, testing standardization, and data interoperability are adequately addressed.

## OCEMs as well-defined platforms for probing structure-performance relationships

A key strength of OCEMs is their ability to construct structurally well-defined and directly comparable model catalytic systems at the single-entity level.^27^^,^^31^ In contrast to conventional powder-based electrodes, these platforms enable a decisive shift in catalyst development: away from heuristic trial-and-error and toward a rational design paradigm supported by precise micro/nanoscale structural control and real-time structure-performance correlations.^32^^,^^33^ This transition provides a robust methodological framework for deciphering complex structure-performance relationships and uncovering detailed reaction mechanisms.^34^^,^^35^ Beyond structural definition, the flexible design and modular fabrication of OCEMs also enable versatile on-device modification strategies.^25^^,^^29^

**Figure 1:**
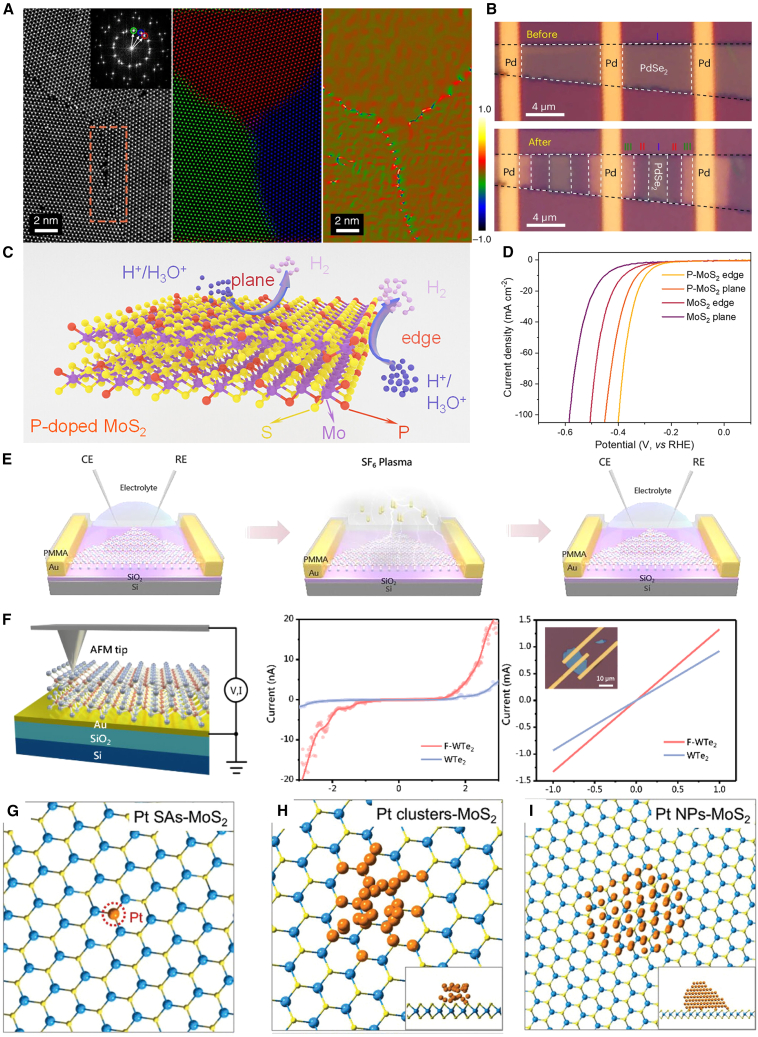
OCEMs as well-defined platforms for constructing model electrocatalytic systems (A) HAADF STEM investigation of MoS_2_ grains. Left: HAADF STEM image showing the GBs between three MoS_2_ grains. Inset on the left figure: Fourier transform of the image showing three distinct sets of MoS_2_ diffraction patterns with rotation angles of 11.6° and 25.6°. Middle: composite color-coded IFFT image. Right: dilatation map after applying GPA routine to the monolayer MoS_2_.^36^ Copyright © 2020 Springer Nature Limited. (B) The device top view showing atomic structures before and after phase engineering. Region I is PdSe_2_ and region II and III are other phases of Pd-Se.^37^ Copyright © 2024 Springer Nature Limited. (C) Schematic comparing the HER performance of the basal plane and edge sites on P-MoS_2_ nanosheets. (D) Polarization curves of the basal plane and edge sites on MoS_2_ and P-MoS_2_ tested in 0.5 M H_2_SO_4_ at a scan rate of 30 mV s^–1.^^32^ Copyright © 2023 American Chemical Society. (E) Schematic diagram of in situ test method. (F) Schematic diagram of CAFM measurement, measured current-voltage characteristic, and in-plane resistance measurement of a WTe_2_ flake before and after plasma processing treatment (optical micrograph of back-gated device shown in inset).^38^ Copyright © 2021 American Chemical Society. (G–I) illustration of the schematic atomic models based on the images of low-Pt catalysts with various Pt loadings on MoS_2_.^39^ Copyright © 2025 Wiley-VCH GmbH.

For instance, the controlled introduction of grain boundaries at the two-dimensional limit yields a well-defined model platform for investigating grain-boundary-mediated electrocatalysis (Figure 1A).^36^ Through on-device phase engineering, the phase structure of a catalyst can not only be tuned in situ but also stabilized in targeted operational states, establishing a versatile platform for correlating phase composition, electronic structure, and catalytic behavior (Figure 1B).^37^ In monolayer MoS_2_ systems, in situ heteroatom doping and defect engineering allowed direct activity comparison on the same nanosheet before and after modification.^32^ This approach revealed that phosphorus doping activates the basal plane and significantly enhances the intrinsic hydrogen evolution reaction (HER) activity of edge sites (Figures 1C and 1D). Such a same-nanosheet comparison is especially powerful for minimizing or eliminating batch-to-batch variations in material synthesis and device assembly.

A comparable single-sheet WTe_2_ electrocatalytic microdevice further demonstrates the reliability of this single-entity platform strategy. Combining in situ SF_6_ plasma treatment with complementary in situ Kelvin probe force microscopy (KPFM) and conductive atomic force microscopy (AFM) characterization^38^ enables direct correlation between the catalytic performance of WTe_2_ and the corresponding shifts in local charge distribution, conductivity, and work function (Figures 1E and 1F).

At a higher level of atomic-scale control, Pt single atoms, clusters, and nanoparticles have been controllably anchored on monolayer MoS_2_ (Figures 1G–1I).^39^ This uniform platform permits direct side-by-side comparison of their catalytic behaviors, enabling the establishment of clear structure-performance relationships across discrete structural motifs from single-atoms to nanoparticles. When combined with high-throughput methods, these targeted modifications can be implemented in parallel across a single catalyst substrate, further reducing experimental variability caused by material inhomogeneity. However, most OCEM studies are proof-of-concept with limited datasets, and challenges in scalability and cross-platform reproducibility persist. Despite these limitations, OCEMs uniquely enable the generation of directly comparable, descriptor-rich datasets suitable for data-driven and ML-guided catalyst discovery. In this context, OCEMs provide a foundation for constructing more consistent and reliable catalyst libraries for accelerated rational catalyst design.

## OCEMs for charge transport, local environment engineering, and catalytic modulation

Beyond their role as well-defined platforms for probing structure-performance relationships, OCEMs offer unique opportunities to actively modulate electrocatalytic behavior through device-level control of charge transport and local reaction environment engineering.^25^^,^^40^^,^^41^ This capability is particularly critical in semiconductor and low-dimensional catalytic systems, where carrier distribution, interfacial electric fields, and local reaction conditions are often tightly coupled to catalytic performance.^42^^,^^43^

**Figure 2:**
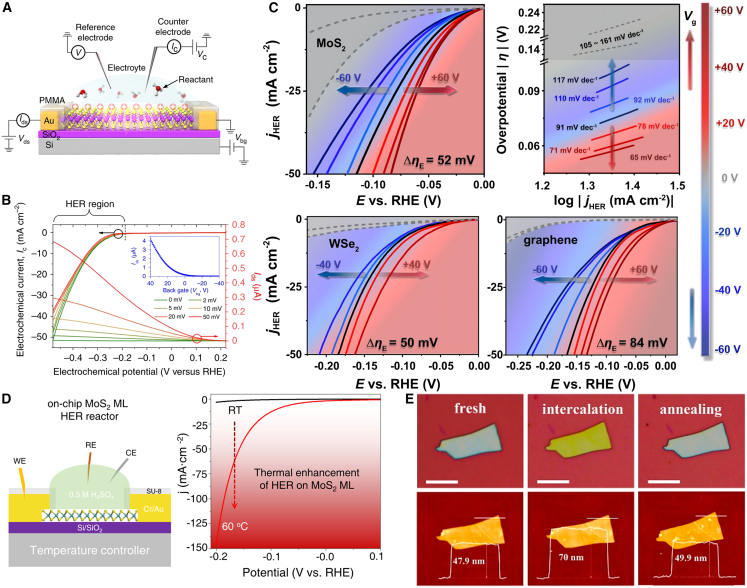
OCEMs enable device-level modulation of charge transport, local reaction environments, and catalytic behavior (A) Schematic of the microcell-based in situ electronic/electrochemical measurements. Both the electronic signal (I_ds_, conductance current) and electrochemical signal (I_c_, reaction current) of the TMD nanosheet can be collected simultaneously. Before the in situ electronic/electrochemical measurements, the type of majority charge carrier for each device was pre-identified by a back-gated measurement (V_bg_) on the SiO_2_ (285 nm)/Si substrate. (B) Optical image of the microcell: overall set-up with four electrodes, enlarged view of the electrolyte (0.5 M H_2_SO_4_) droplet in which the reaction occurs, and the mechanically exfoliated TMD device with a micro-sized reaction window in the PMMA passivation.^42^ Copyright © 2019 Springer Nature Limited. (C) HER polarization curves and Tafel curves of Pt SAs-MoS_2_ in response to OEEFs. HER polarization curves of Pt SAs-WSe_2_ and Pt SAs-graphene in response to OEEFs. The two gray dashed lines represent the tunable HER activity of pristine 2D substrates under OEEFs modulation. The color bar shows the direction and scope of Vg.^43^ Copyright © 2022 Springer Nature Limited. (D) Cross section of the on-chip measurement setup, and polarization curves of the MoS_2_ ML obtained from room temperature to 60°C.^44^ Copyright © 2022 American Chemical Society. (E) Optical micrographs and AFM for the same TaS_2_ nanosheet corresponding to fresh, intercalated, and annealing states. Scale bars, 10 μm.^45^ Copyright © 2020 American Chemical Society.

The OCEMs architecture enables modes of modulation unattainable with conventional electrochemical setups. For instance, the self-gating phenomenon and the leakage-current metal-insulator-semiconductor (LMIS) model for semiconductor electrocatalysis explain reaction-induced modulation of the surface Fermi level and conductance.^42^ This study clarified the transport behavior associated with the coexisting active and inactive regions in semiconductor catalysts, leveraging OCEMs to establish clear correlations (Figures 2A and 2B). It further delineated relationships among carrier type, reaction type, and the penetration depth of the highly conductive surface layer, providing a new electron-transport framework for understanding the high activity of nanostructured semiconducting catalysts. Inspired by electrostatic regulation in enzymatic catalysis, oriented external electric fields (OEEFs) have emerged as an effective strategy for modulating catalytic activity and selectivity in single-molecule reactions. This concept was extended to single-atom catalytic systems through directional external electric-field control,^43^ enabling continuous, quantitative, and dynamic modulation of the local reaction environment around single-atom sites. This work demonstrated that local electric fields can tune the interaction between frontier orbitals and reaction intermediates, lowering the barrier of the rate-determining step, and thereby offering a pathway to transcend volcano-type constraints (Figure 2C).

Beyond electronic state modulation, local reaction environment engineering has expanded to controlling thermal effects, structural states, and strain. For example, studies using an on-chip monolayer MoS_2_ reactor to isolate thermal contributions showed that temperature control can optimize electron-transfer processes both within atomically thin materials and across the solid-liquid interface, thereby improving HER performance (Figure 2D).^44^ Similarly, a molecular intercalation strategy was used to construct TaS_2_-N_2_H_4_ two-dimensional hybrid superlattices, achieving interlayer expansion, in-plane strain modulation, and directional charge transfer. These local reconstructions markedly accelerated the kinetics of HER (Figure 2E).^45^

Collectively, these studies illustrate that OCEMs are not merely passive testing devices but an active platform that enables the coupling of charge transport, local reaction environment, and catalytic kinetics within a single experimental framework. By enabling controlled modulation of electronic states, local electric fields, thermal effects, and strain, OCEMs provide a versatile platform for probing electrocatalytic behavior. These capabilities allow researchers to elucidate how charge transport and local environment engineering collectively govern catalytic activity, laying the foundation for developing more precise catalyst design strategies. However, translating device-level control into practical high-throughput systems remains challenging, particularly for complex multistep reactions. Future OCEMs work must balance single-device mechanistic clarity with scalable, statistically robust platforms.

## OCEMs as in situ/operando platforms for mechanistic studies

**Figure 3:**
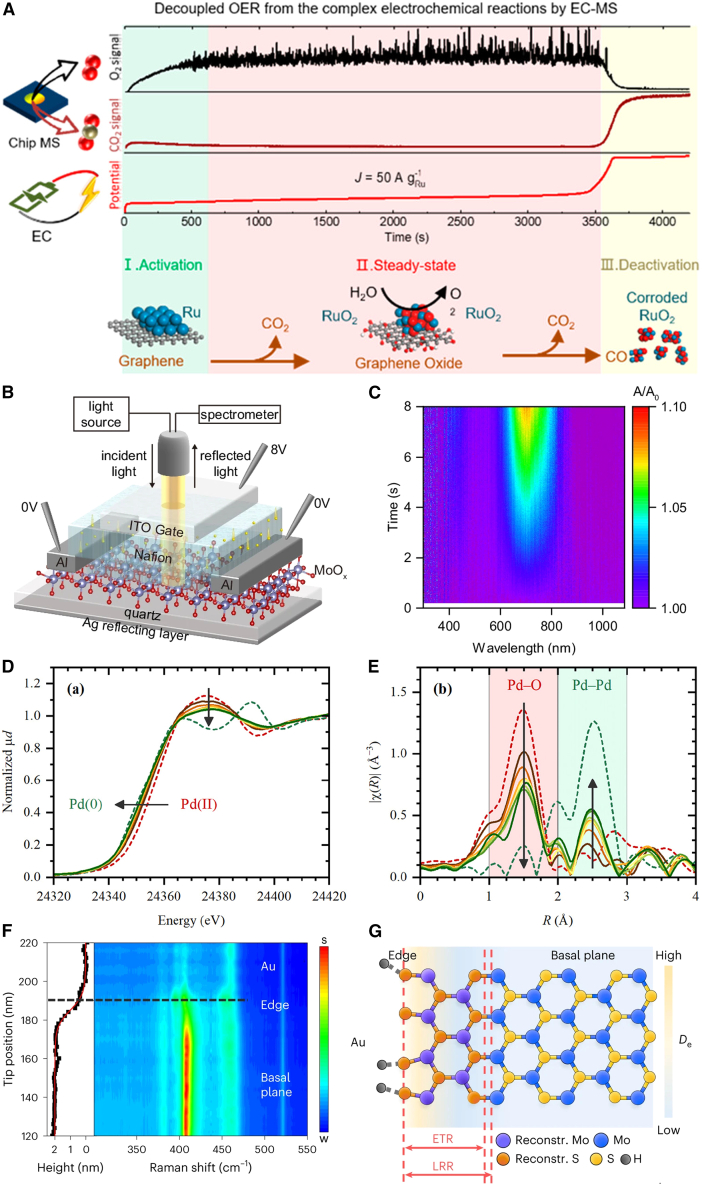
OCEM integrates in situ/operando multimodal characterization for tracking dynamic structural evolution and active-site behavior (A) EC-MS experiments using chronopotentiometry (CP) technique in 0.05 M H_2_SO_4_. The bottom curves were the electrode potential as a function of time at a constant current of 50 A g^−1^_Ru_. The loaded Ru mass was about 0.0119 mg Ru cm^−2^ for the Ru/G-450 Red catalyst. The middle and the top curves showed the corresponding Faraday efficiency for CO_2_ evolution (FE-CO_2_) and O_2_ evolution (FE-O_2_) based on MS signals of m/z = 44 and m/z = 32.^47^ Copyright © 2021 American Chemical Society. (B) Schematic of experimental setup used for in situ real-time optical absorption measurement. (C) The time-resolved UV-vis absorption mapping.^49^ Copyright © 2025 Springer Nature Limited. (D and E) (D) Evolution of in situ XANES and (E) FT-EXAFS data collected in different channels of the microchip (solid lines, from brown to green). Dashed red and green lines corresponded to the reference palladium (II) acetate and palladium foil, respectively. The FT-EXAFS spectrum of Pd foil in part (E) was scaled by 0.5 for visualization purposes.^50^ Copyright © 2023 American Chemical Society. (F) EC-TERS line image across the edge of a bilayer MoS_2_. Left: topographic height profile of the edge. Right: color-coded intensity map of the line-trace TERS spectra across the edge at −0.15 V versus RHE. The color scale bar denoted the TERS intensity from weak (w) to strong (s). Note: these data were original TERS experimental spectra without any treatment. (G) Schematic diagram of the LRR and ETR near the edge. D_e_, electron density.^51^ Copyright © 2024 Springer Nature Limited.

The highly integrated, open architecture of OCEMs allows for deep coupling with diverse characterization techniques, facilitating in situ/operando analysis of electrochemical reactions and thus establishing a more direct correlation between catalytic behavior and structural evolution.^46^^,^^47^^,^^48^ More recently, OCEMs platforms have been extended to probe reaction-coupled phase transitions. For example, an on-chip electrochemistry-mass spectrometry system enables rapid sampling and highly sensitive mass spectrometry detection of the oxygen evolution reaction (OER) on graphene-supported Ru catalysts, achieving real-time product monitoring at the pmol s^−1^ level (Figure 3A).^47^ Similarly, operando electrical and spectral measurements on a MoO_x_ device revealed threshold-driven proton dynamics that switch the system from conductance modulation to catalytic HER activity, directly linking proton intercalation to reaction behavior (Figures 3B and 3C).^49^

Beyond product detection, OCEMs are also well suited for integrating with high-resolution structural characterization techniques to track in situ structural changes during reactions. By combining operando hard X-ray ptychography with electron microscopy, researchers have observed internal structural evolution and deactivation processes of catalysts, such as silver-catalyzed methanol oxidation, under realistic reaction environments.^52^ Integrating X-ray absorption spectroscopy with microfluidic platforms provides an effective approach for in situ/operando characterization under reaction conditions (Figures 3D and 3E).^50^ Directly coupling the X-ray beam with microfluidic channels enables high-quality spectral acquisition and time-resolved tracking of metal species evolution, as demonstrated in the monitoring of Pd nanoparticle nucleation.

Furthermore, the combination of OCEMs with high-spatial-resolution spectroscopy enables refined characterization of active-site atomic structures. Specifically, using electrochemical tip-enhanced Raman spectroscopy (EC-TERS), researchers directly observed that during the HER process, edge regions of MoS_2_ undergo coupled lattice and electronic reconstruction during electrochemical activation (Figures 3F and 3G).^51^ Collectively, this high spatiotemporal resolution capability shifts the focus from large-scale, multi-site averaged signals to site-specific structural evolution during reactions, providing unprecedented precision for investigating structure-performance relationships and active-site mechanisms.

Overall, these developments continue to expand the structural and functional scope of OCEM-based studies. Integrating OCEMs with high-throughput methods holds great potential for elucidating electrocatalyst active-site mechanisms and structure-performance relationships, while also offering more diverse modification strategies to advance rational catalyst design. However, current operando OCEMs remain limited by low throughput and fragmented data, which hinder systematic comparison across different systems. Notably, OCEMs also offer a unique opportunity to standardize time- and spatially resolved electrochemical measurements into machine-processable datasets, providing a critical foundation for data-driven catalyst discovery.

## OCEMs as potential platforms toward HTE and data-driven discovery

**Figure 4:**
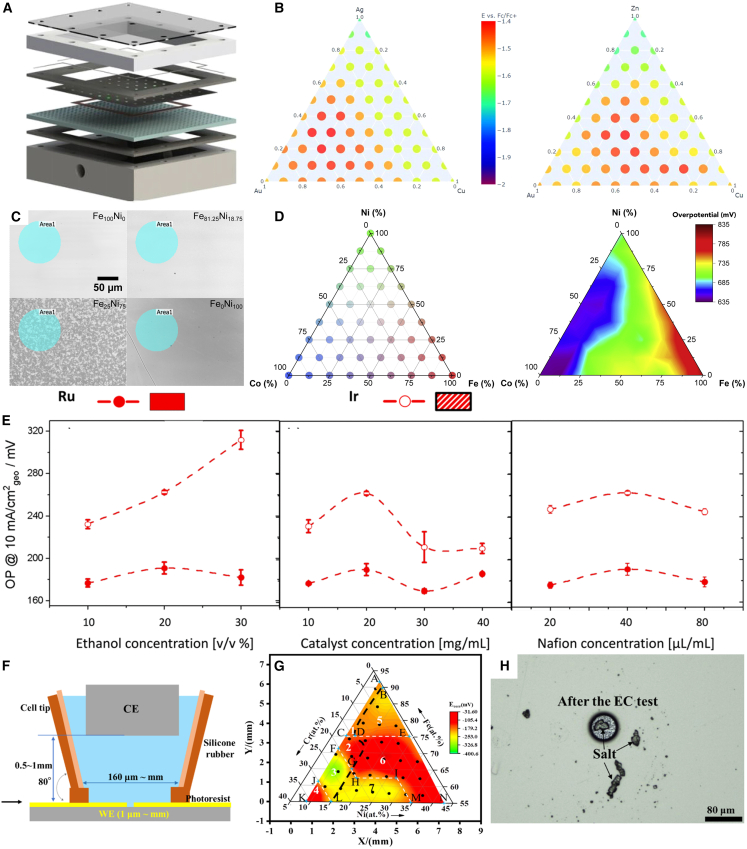
Automated and combinatorial high-throughput electrochemical platforms for catalyst screening and mechanistic evaluation (A) Exploded view of the electrochemical screening cell. Pt and Ag wires are used as the counter and reference electrodes, respectively. A Cu current collector is beneath the array working electrode. The cell was sandwiched between silicone gaskets and isolated from air. (B) Screening experiments were performed with 1 M ionic liquid and 0.5 M DI water in acetonitrile. In total, 66 Au–Ag–Cu and Au–Zn–Cu compositions screened under CO_2_.^55^ Copyright © 2021 Springer Nature Limited. (C) Representative close-up images of the Fe-Ni library obtained during the quality assurance step with the laser microscope in teaching mode. The scale bars correspond to 50 μm. (D) Activity of Fe-Ni-Co oxide library in 0.05 M KPi at 5 mA cm^−2^. Ternary diagram of the synthesized Fe-Ni-Co oxide library, and contour plot of overpotential mapped over the ternary diagram.^56^ Copyright © 2022 Elsevier Inc. (E) Impact of varying ink formulation parameters on OER performance for Ru and Ir metals. A set of experiments showing the effect of varying ethanol concentration, catalyst concentration (mass loading), and Nafion concentration on the overpotential (OP).^57^ Copyright © 2024 Royal Society of Chemistry. (F) Enlarged display of the mini-cell construction. (G) Composition-phase-E_corr_ mapping of the iron-based alloy in the Fe-Cr-Ni combinatorial materials chip. (H) Metallographic observation of the sample covered with photoresist after electrochemical test.^58^ Copyright © 2021 The Electrochemical Society.

HTE has emerged as a powerful strategy for electrocatalyst discovery, enabling rapid, parallel screening of materials.^35^^,^^53^^,^^54^ Integrating automation with HTE yields efficient, reliable experimental workflows that substantially reduce manual labor and supervision. When coupled with automation, these platforms enable standardized, parallelized, and reproducible testing, significantly improving experimental efficiency and data consistency capabilities validated across multiple research fields.^55^^,^^56^ Some of the platforms shown in the figures are non-OCEM high-throughput systems. They are included here to illustrate methodological strategies, such as automation, combinatorial library construction, and multi-parameter screening, which may inspire future OCEM-based HTE designs, rather than representing current OCEM capabilities. For instance, although not based on OCEMs architecture, multi-metal catalyst arrays were deposited on carbon paper, and a 3D-printing-based high-throughput platform was established to screen CO_2_ reduction reaction (CO_2_RR) catalysts.^55^ This approach identified Au_6_Ag_2_Cu_2_ and Au_4_Zn_3_Cu_3_ as the best ternary catalysts in the library (Figures 4A and 4B), demonstrating screening principles that could be transferred to OCEMs platforms. Moreover, an automated scanning flow cell coupled with inductively coupled plasma mass spectrometry enabled simultaneous determination of OER catalyst activity and stability.^56^ Through automated high-throughput screening, Fe-Ni-Co oxides were found to achieve superior activity-stability synergy compared to Fe-Ni catalysts (Figures 4C and 4D).

A key direction in HTE is the construction of catalyst libraries that enable systematic mapping of structure-performance relationships. In such libraries, catalyst performance is correlated with well-defined structural features, which can be probed by in situ/operando characterization, facilitating data-driven screening of highly efficient catalysts. For example, the automated, modular electrochemical testing platform (AMPERE) enables rapid and reproducible screening by standardizing experimental procedures and minimizing human intervention. Using this platform, 168 experiments were completed within 40 h to systematically investigate how ink formulation influences the OER performance of Ir, Ru, IrO_2_, and RuO_2_ catalysts in acidic media.^57^ The results showed that improved activity of Ru/RuO_2_ catalysts was neither directly associated with increased electrochemical surface area nor inversely correlated with Ru dissolution (Figure 4E), highlighting the ability of automated platforms to decouple multiple variables.

Beyond rapid performance screening, automated platforms can be integrated with stability and degradation analysis. For instance, an automated electrochemical testing platform was developed, incorporating a micro-electrochemical cell, automatic liquid refresh system, programmed scanning XYZ stage, and control system.^58^ Coupled with in situ inductively coupled plasma mass spectrometry (ICP-MS), this platform was used to investigate the corrosion behavior of Fe-Cr-Ni alloys with varying compositions during HER (Figures 4F–4H). Importantly, this integrated system enables rapid and parallel evaluation of both catalytic performance and stability across compositional libraries, demonstrating the capability of automated platforms for high-throughput and multidimensional catalyst assessment.

Despite advances in HTE, constructing catalyst libraries imposes stringent demands on high-throughput platforms for catalyst screening and mechanistic studies, requiring simultaneous achievement of data richness and high reproducibility.^10^^,^^56^ However, many existing HTE approaches generate large datasets that lack mechanistic interpretability, limiting their ability to establish causal structure-performance relationships. In this context, OCEMs provide structurally well-defined, descriptor-rich, and mechanistically interpretable platforms, enabling the transition from correlative screening to causal understanding of catalytic behavior. Nevertheless, this limitation reflects a broader bottleneck in current high-throughput methodologies.^59^ In this context, an ideal high-throughput platform for electrocatalysis should not only enable rapid and parallel testing but also provide structurally controllable catalyst systems and compatibility with mechanistic characterization.

OCEMs address this need by offering a promising platform for high-throughput electrocatalysis, leveraging structurally controllable catalyst systems and highly integrated device architectures. Their on-chip design allows configuration into arrayed formats, making them well suited for automated HTE. By integrating two-dimensional materials into well-defined device architectures, OCEMs enable precise control over catalyst structures at the single-entity level, including active sites,^51^ phase states,^37^ and defect configurations.^60^ This capability allows direct comparison of different catalytic configurations on the same substrate, minimizing batch-to-batch variations and improving reproducibility. Additionally, advances in on-chip fabrication and automated testing strategies enable parallel preparation and evaluation of catalyst arrays, facilitating standardized, efficient catalyst design and development.

**Figure 5:**
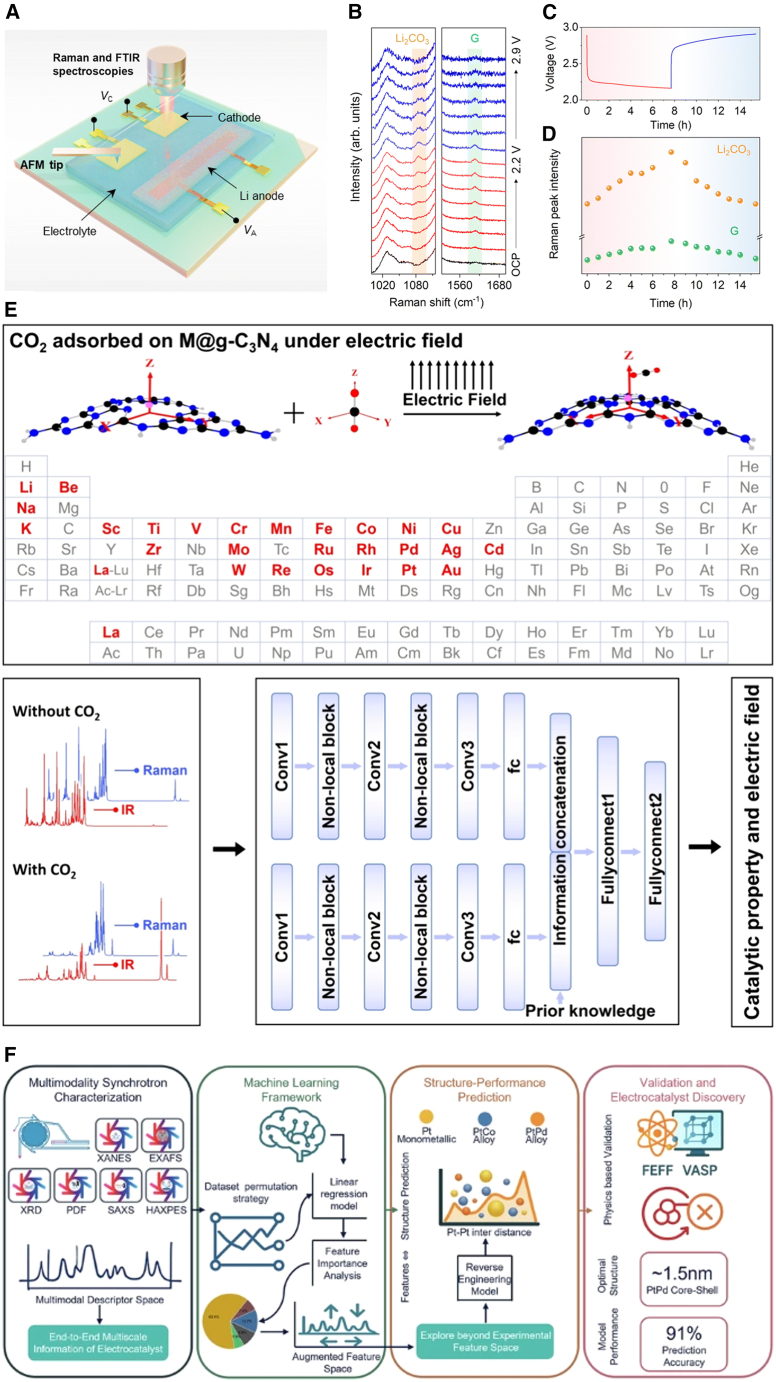
Emerging OCEM-based opportunities for high-throughput, operando, and machine-learning-assisted electrocatalyst discovery (A) Schematic illustration of the designed on-chip LCB array with in situ EC-Raman spectroscopy and EC-AFM functionality. (B) EC-Raman spectra of the Pt cathode recorded during the corresponding galvanostatic discharge-charge process. Black, red, and blue curves represent the battery in the open-circuit potential (OCP) state, the discharge state, and the charge state, respectively. (C) Galvanostatic discharge-charge test of the Pt-based on-chip LCB at a constant current of 2 μA. (D) Raman peak intensity changes of Li_2_CO_3_ (∼1085 cm^−1^) and carbon (G band, ∼1604 cm^−1^) during the corresponding discharge–charge process.^20^ Copyright © 2023 Royal Society of Chemistry. (E) Flowchart for predicting the adsorption energy of M@*g*-C_3_N_4_ for carbon dioxide through machine learning.^21^ Copyright © 2024 American Chemical Society. (F) Schematic illustration of machine learning-guided multimodal synchrotron analysis workflow for data-driven fuel cell electrocatalyst discovery.^22^ Copyright © 2025 Springer Nature Limited.

For example, a multifunctional on-chip Li-CO_2_ battery (LCB) testing platform was developed to investigate reaction processes and product formation during electrochemical conversion (Figures 5A–5D).^20^ Pt, Au, Ag, Cu, Fe, and Ni nanoparticles were deposited via electron beam deposition and evaluated on the LCB platform, demonstrating potential for parallel HTE. In situ electrochemical Raman (EC-Raman) spectroscopy and electrochemical atomic force microscopy (EC-AFM) measurements were further conducted on the LCB platform, revealing that only Li_2_CO_3_ and carbon species formed during discharge process in Pt-based on-chip LCBs. This example underscores how the open architecture of OCEMs facilitates integration with in situ/operando characterization, enabling simultaneous mechanistic studies and HTE.

At the present stage, the integration of ML with OCEMs should be regarded as an emerging opportunity rather than a fully established capability. A realistic ML-assisted OCEM workflow would require four linked components: standardized on-chip catalyst libraries, high-throughput electrochemical readouts, time-synchronized in situ/operando descriptors, and model-guided feedback for selecting the next experimental conditions. In this workflow, OCEMs are not merely miniaturized testing cells; they can provide spatially resolved and device-indexed datasets that connect catalyst configuration, local reaction environment, operando spectral features, and catalytic performance. These datasets could then be used to train surrogate models, graph-based models, or Bayesian optimization frameworks for identifying activity-stability-cost trade-offs and for prioritizing new catalyst compositions or site configurations.

Recent data-driven studies provide useful methodological references for developing ML-assisted OCEM platforms, although such workflows have not yet been fully implemented in OCEMs. For example, multiobjective Bayesian optimization has been used to explore high-entropy alloy electrocatalysts in vast composition spaces by simultaneously considering catalytic activity, cost effectiveness, and entropic stabilization.^61^ In addition, recent advances in data-driven dual-atom catalyst design show that high-throughput calculations and ML can correlate dual-site configurations, coordination environments, electronic descriptors, and catalytic performance.^62^ In a complementary approach, spectral features derived from infrared/Raman signals of CO_2_ molecules can be used to extract descriptors related to adsorption energy and charge transfer. This strategy has been applied to investigate CO_2_ adsorption on 27 different metal single atoms supported on graphitic carbon nitride (g-C_3_N_4_) under varying electric field directions and strengths (Figure 5E).^21^ Combining these spectral features with ML enables the establishment of a spectral property model linking infrared/Raman spectra to adsorption energy and charge transfer, facilitating quantitative evaluation of electric field effects on CO_2_ catalytic conversion. An ML model for structure-performance prediction was developed, utilizing descriptors derived from in situ/operando spectral characterization (Figure 5F).^22^ In situ/operando techniques including X-ray absorption near-edge structure (XANES), extended X-ray absorption fine structure (EXAFS), X-ray diffraction (XRD), small-angle X-ray scattering (SAXS), pair distribution function (PDF) analysis, and hard X-ray photoelectron spectroscopy (HAXPES) were employed for end-to-end multiscale measurements across a diverse range of fuel cell catalysts. The ML models predict key catalytic performance metrics, including mass activity, specific activity, and electrochemical surface area with high accuracy. This interpretable model goes beyond black-box prediction to provide physical insights into the relationships between structural features, measured spectra, and catalytic behavior. It also clarifies how physically meaningful descriptors from in situ/operando spectroscopy and theoretical calculations relate to catalytic activity, surface area, and local structure. These studies suggest that the future role of OCEMs may lie in supplying experimentally standardized, descriptor-rich datasets that complement computational screening and enable more reliable model validation. Representative OCEM and high-throughput electrocatalysis platforms, together with their catalyst systems, modulation strategies, in situ/operando characterization methods, and reported electrocatalytic performance, are summarized in Table 1.

**Table 1:** Summary of OCEM platforms, catalyst samples, modulation strategies, in situ/operando characterization, and electrocatalytic performance

Catalyst Samples	Active structure	Modulation strategy	In situ/operando characterization	Performance	Reference
MoS_2_ GBs	grain boundaries	Au QD	–	HER −25 mV 54 mV dec^−1^	He et al.^36^
PdSe_2_	Pd_17_Se_15_/Pd_4_Se	thermal phase engineering	–	HER 44 mV dec^−1^	Liu et al.^37^
P-MoS_2_	heteroatom doping edge	phosphorus doped	–	HER −30 mV 48 mV dec^−1^	Wang et al.^32^
1T′-WTe_2_	basal plane	F doped	In situ Raman	HER −270 mV 173 mV dec^−1^	Yang et al.^38^
Pt-MoS_2_	Pt SAs/clusters/NPs	defect anchoring	–	HER −94 mV 65.9 mV dec^−1^	Yan et al.^39^
WS_2_/MoS_2_	basal plane	self-gating	In situ electronic/electrochemical measurements	HER −208 mV 108 mV dec^−1^	He et al.^42^
Pt SAs-MoS_2_	Pt SAs	defect anchoring	In situ electronic/electrochemical measurements	HER −20 mV 51 mV dec^−1^	Pan et al.^43^
1T-MoS_2_	basal plane	mechanical exfoliation	In situ electronic/electrochemical measurements	HER −90 mV 94 mV dec^−1^	Qu et al.^44^
TaS_2_-N_2_H_4_	N_2_H_4_-intercalated TaS_2_ hybrid superlattice	spontaneous hydrazine; molecular intercalation	In situ electronic/electrochemical measurements In situ KPFM/Raman	HER 450 mV (38.6 mA cm^−2^) 76 mV dec^−1^	Guo et al.^45^
MoO_x_	M-*O*-H sites	ALD, proton intercalation	In situ electrical/optical measurements	HER 60 mV dec^−1^	Liang et al.^49^
Pd NPs	Pd (0) nanoparticles/clusters	3D-printed PETG	In situ K-edge XAS/XANES/EXAFS	–	Dobrovolskaya et al.^50^
Fe-Cr-Ni	Fe-based alloy	combinatorial ion beam deposition	In situ electronic/electrochemical measurements	–	Lai et al.^58^
Pt-based LCB	Pt (111)	E-beam deposition	In situ EC-Raman/EC-FTIR/EC-AFM	Li-CO_2_ battery η ≈ 0.55 V EE ≈ 80–90%	Wang et al.^20^

Nevertheless, several challenges must be addressed before closed-loop ML-guided OCEM discovery becomes practical. Current OCEM studies are still dominated by single-device demonstrations or small device arrays, resulting in limited dataset size and insufficient cross-device statistics. In addition, differences in electrode geometry, catalyst loading, electrolyte configuration, reference electrode calibration, and operando data synchronization can introduce nontrivial batch effects. Therefore, future efforts should focus on establishing unified device metadata, standardized electrochemical protocols, machine-readable operando spectral formats, and uncertainty-aware model validation. Only with these foundations can OCEMs evolve from mechanistic model platforms into reliable experimental infrastructures for data-driven and ML-assisted electrocatalyst discovery.

## Conclusion and outlook

In summary, OCEMs are promising candidates for developing multifunctional high-throughput electrocatalysis platforms, owing to their open architecture, array compatibility, controllable catalyst configurations, and compatibility with in situ/operando characterization. These inherent features enable parallel catalyst testing, standardized reaction environments, and automated data acquisition, providing a viable pathway to address long-standing challenges in conventional catalyst evaluation, including low screening efficiency, poor comparability, and insufficient reproducibility.

The significance of OCEMs lies not only in their ability to construct well-defined electrocatalytic model systems, which facilitate the exploration of structure-performance relationships, but also in their potential to further evolve into high-throughput experimental platforms. From a high-throughput perspective, the greatest promise of OCEMs extends beyond faster catalyst screening to the generation of high-quality electrocatalytic data with mechanistic depth. OCEM-based platforms integrate screening with in situ/operando characterization, allowing catalytic activity to be interpreted alongside structural evolution, local reaction environments, intermediate formation, and deactivation behavior. Building on these capabilities, OCEMs enable the generation of mechanistically informed datasets that couple catalytic performance with structural dynamics, local environments, intermediate species, and degradation pathways.

Such datasets can be translated into quantitative features derived from in situ/operando characterization to construct physically meaningful descriptors, laying the groundwork for catalyst databases that support data-driven catalyst discovery. Furthermore, coupling high-throughput OCEM platforms with ML may provide a future route for uncovering hidden structure-performance relationships, provided that OCEM-generated datasets become sufficiently standardized, reproducible, and machine-processable. Rather than representing a mature closed-loop discovery platform at present, OCEMs should be viewed as a promising experimental foundation for building mechanism-informed datasets that could support future ML-guided catalyst design. In this regard, the future perspective of OCEMs may lie in driving electrocatalysis research away from low-throughput empirical exploration toward a more automated, mechanism-oriented, and data-driven paradigm.

First, the fabrication of OCEM devices largely relies on highly stochastic processes such as exfoliation of small-sized nanosheets and manual assembly. Batch-to-batch consistency is difficult to guarantee, fabrication efficiency is low, and yield stability is poor. To achieve truly potential HTE platforms, it is essential to develop automated and standardized OCEM manufacturing procedures, as well as wafer-scale material preparation and transfer, in order to ensure consistency in experimental workflows, experimental conditions, and substrate materials. This requires the establishment of standardized fabrication protocols compatible with automated production and testing.

Second, current OCEM demonstrations are mostly focused on reactions such as OER and HER. For more diverse reaction systems (e.g., NRR, NO_3_RR), stringent demands are placed on the sealing design, mass-transport design, and working electrode design of OCEMs. In particular, when integrating with microfluidic electrochemical cells, it is foreseeable that OCEMs intended for HTE will encounter significant difficulties in both design and fabrication.

Furthermore, although OCEMs are designed to support automated data acquisition, there is a lack of unified standards across laboratories regarding electrode design, electrochemical testing protocols, and even performance presentation methods. As a result, the generated datasets are difficult to compare directly. Notably, in many existing OCEMs intended for HTE, a considerable portion of material performance is initially characterized through non-data-driven means such as bubble measurement, followed by further testing using conventional electrochemical methods. This can lead to relatively coarse measurement results and preclude cross-device comparison. Establishing standardized testing protocols, such as specifying reference electrode types and current normalization methods (geometric area vs. real specific surface area), is a prerequisite for building reproducible and shareable catalyst databases. Moreover, the integration with in situ/operando characterization techniques (e.g., Raman, X-ray absorption spectroscopy, DEMS) suffers from a lack of industry consensus on data formats, time synchronization, and processing norms, which limits the capability of high-throughput platforms to generate machine-processable data.

Overcoming these barriers is essential to transform OCEMs from powerful mechanistic tools into practical infrastructures for high-throughput electrocatalyst screening and discovery. The key value lies in enabling a unified framework that integrates mechanistic understanding directly into high-throughput workflows, thereby shifting catalyst development from empirical screening toward mechanism-informed and physics-guided discovery. In this paradigm, OCEMs generate physically interpretable datasets that are compatible with autonomous experimentation and ML-guided materials design. Although OCEMs are unlikely to replace all conventional electrocatalytic platforms, they represent one of the most promising pathways toward high-throughput, mechanism-rich, and data-driven electrocatalyst discovery.

## Acknowledgments

This work was supported by the 10.13039/501100001809National Natural Science Foundation of China (grant number: 52371229).

## Author contributions

Z.L. conceived the study and wrote the original draft. J.T. and G.O. performed the investigation and data curation. R.C. and C.L. reviewed and edited the manuscript. L.J. acquired funding, administered the project, and provided supervision. All authors discussed the results and approved the final manuscript.

## Declaration of interests

The authors declare no competing interests.

## Declaration of generative AI and AI-assisted technologies in the writing process

During the preparation of this manuscript, the authors used ChatGPT (OpenAI) solely for language polishing, grammar refinement, and improving readability. No AI-assisted tools were used to generate scientific ideas, analyze data, draw conclusions, or create original research content. The authors reviewed and edited all AI-assisted outputs and take full responsibility for the final content of the manuscript.
